# A “Timed” Kiss Is Essential for Reproduction: Lessons from Mammalian Studies

**DOI:** 10.3389/fendo.2016.00121

**Published:** 2016-08-31

**Authors:** Manish Putteeraj, Tomoko Soga, Takayoshi Ubuka, Ishwar S. Parhar

**Affiliations:** ^1^Brain Research Institute (BRIMS), Jeffrey Cheah School of Medicine and Health Sciences, Monash University Malaysia, Petaling Jaya, Malaysia

**Keywords:** kisspeptin, reproduction, circadian rhythms, clock genes, GnRH, AVPV

## Abstract

Reproduction is associated with the circadian system, primarily as a result of the connectivity between the biological clock in the suprachiasmatic nucleus (SCN) and reproduction-regulating brain regions, such as preoptic area (POA), anteroventral periventricular nucleus (AVPV), and arcuate nucleus (ARC). Networking of the central pacemaker to these hypothalamic brain regions is partly represented by close fiber appositions to specialized neurons, such as kisspeptin and gonadotropin-releasing hormone (GnRH) neurons; accounting for rhythmic release of gonadotropins and sex steroids. Numerous studies have attempted to dissect the neurochemical properties of GnRH neurons, which possess intrinsic oscillatory features through the presence of clock genes to regulate the pulsatile and circadian secretion. However, less attention has been given to kisspeptin, the upstream regulator of GnRH and a potent mediator of reproductive functions including puberty. Kisspeptin exerts its stimulatory effects on GnRH secretion *via* its cognate Kiss-1R receptor that is co-expressed on GnRH neurons. Emerging studies have found that kisspeptin neurons oscillate on a circadian basis and that these neurons also express clock genes that are thought to regulate its rhythmic activities. Based on the fiber networks between the SCN and reproductive nuclei such as the POA, AVPV, and ARC, it is suggested that interactions among the central biological clock and reproductive neurons ensure optimal reproductive functionality. Within this neuronal circuitry, kisspeptin neuronal system is likely to “time” reproduction in a long term during development and aging, in a medium term to regulate circadian or estrus cycle, and in a short term to regulate pulsatile GnRH secretion.

## Introduction

**Figure 1:**
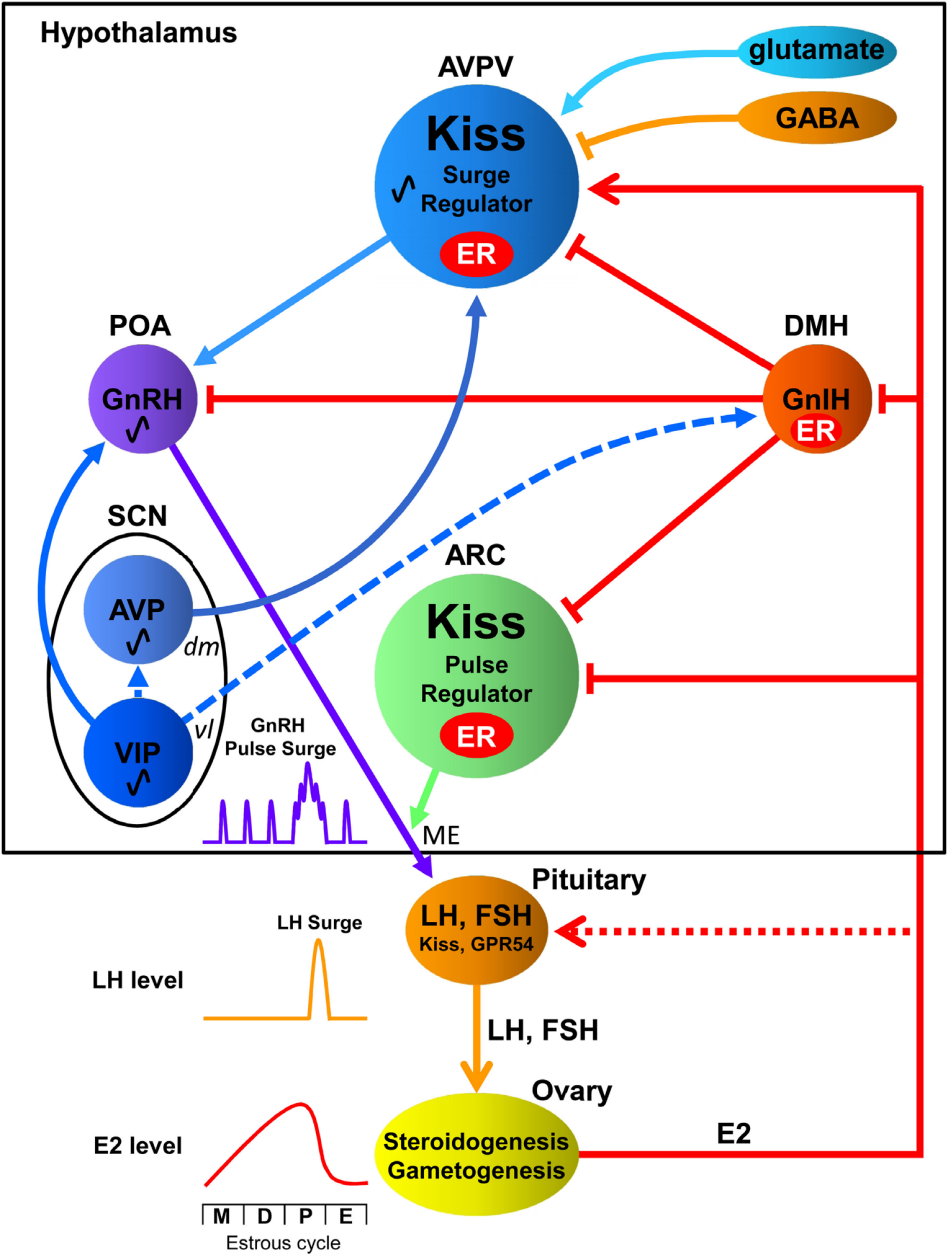
**Interactions among the central biological clock and reproductive neurons in the hypothalamic–pituitary–gonadal axis of females**. The suprachiasmatic nucleus (SCN), the central biological clock, can be divided into two major subdivisions known as the ventrolateral (*vl*) SCN, the core, and the dorsomedial (*dm*) SCN, the shell. The former contains cell bodies of vasoactive intestinal polypeptide (VIP) neurons and the latter contains cell bodies of arginine vasopressin (AVP) neurons. The *vl*SCN acts as the conductor of rhythmicity and transmits synchronizing cues to the *dm*SCN. VIP neurons project to gonadotropin-releasing hormone (GnRH) neurons in the preoptic area (POA), whereas AVP neurons project to kisspeptin (Kiss) neurons in the anteroventral periventricular nucleus (AVPV). Gonadotropin-inhibitory hormone (GnIH) neurons in the dorsomedial hypothalamus (DMH) inhibit the activity of GnRH neurons as well as kisspeptin neurons in the AVPV and arcuate nucleus (ARC). AVPV kisspeptin neuron is also regulated by stimulatory and inhibitory neurotransmitters glutamate and GABA, respectively. GnRH is released at the median eminence (ME) to stimulate luteinizing hormone (LH) and follicle-stimulating hormone (FSH) secretion from the pituitary, which stimulate steroidogenesis and gametogenesis in the ovary. Estradiol (E2) secreted from the ovary gradually increases and rapidly decreases during the estrous cycle [metestrous (M), diestrus (D), proestrus (P), and estrus (E)] in rodents. GnIH and Kiss neurons express estrogen receptor (ER) to convey hormonal information to the reproductive neuronal network. ARC Kiss neurons may function as part of the negative feedback mechanism of E2 on pulsatile GnRH release at the ME. On the other hand, AVPV Kiss neurons may function as the positive feedback mechanism of high E2 concentration on GnRH/LH surge. E2 inhibits GnIH gene expression in the DMH and Kiss gene expression in the ARC but stimulates Kiss gene expression in the AVPV. Kiss and GPR54 are reported to be expressed in gonadotropes, and they are thought to exert synergic effects with GnRH and E2 on LH release (13). Solid lines indicate direct regulation by receptors of signaling molecules, whereas dotted lines indicate possible indirect regulation.

Reproduction, a central feature of life, requires synergistic actions of cellular processes at the brain and the reproductive organs to achieve normal sexual functionalities. Several neuronal populations in the hypothalamus including preoptic area (POA), anteroventral periventricular nucleus (AVPV), and arcuate nucleus (ARC) play critical roles in the hypothalamic–pituitary–gonadal (HPG) axis (1–4). Importantly, POA contains gonadotropin-releasing hormone (GnRH) neurons and AVPV and ARC contain a distinct neuronal population termed kisspeptin neurons; which are fundamental in the feedback system of the HPG axis (5–9). When kisspeptin is bound to its cognate G-protein-coupled receptor (GPR) 54, commonly known as *Kiss1-R*, to stimulate GnRH secretion, the release of luteinizing hormone (LH) and follicle-stimulating hormone (FSH) from gonadotropes occurs (6). This process culminates in the synthesis of sex steroids at the gonads and, in concert with the action of gonadotropins, stimulates gametogenesis (10–12) (Figure 1).

Gonadotropin-releasing hormone neurons exhibit pulsatile and surge secretory patterns in females, with peaks occurring prior to the LH surge ensuring the temporal regulation of the HPG axis. Rhythmicity of GnRH neuronal activity and reproductive hormonal fluctuation are studied well in female rodents. During their estrous cycle (metestrous, diestrus, proestrus, and estrus), which normally cycles in 4–5 days in rats and mice, estradiol secreted from the ovary gradually increases until proestrus stage and rapidly decreases at estrus stage. Relatively low concentration of estradiol during metestrous and diestrus stages inhibits GnRH pulsatility. However, high concentration of estradiol in the afternoon of proestrus stage increases the frequency and amplitude of GnRH pulsatility resulting in GnRH/LH surge that induces ovulation from the ovary (14, 15) (Figure 1).

The HPG axis does not operate independently and is gated by multiple neurocircuitries, one of which is the circadian clock system. The term “circadian” is derived from the Latin words “circa” and “dies” referring to “around a day”; hence, the circadian clock system ensures the timely regulation of physiological and molecular processes over a 24-h cycle. The cyclic features of reproduction are the result of synchrony to the circadian system. The importance of the circadian system in the regulation of the HPG axis was clearly shown by GnRH neuron firing activity of ovariectomized mice treated with high concentration of estradiol implants (OVX + E). Increased GnRH neuron firing activity and LH surge only occurred in the late afternoon or early night in OVE + E mice (16). Reproductive behavior is also regulated by the circadian system, such as increased sexual desire evident in men during the morning phase triggered by high testosterone levels (17–20). Similarly, in rodent species, female mice appear to be more sexually receptive during the early night, a behavior associated with the circadian profile of gonadotropins and sex steroid release (21, 22).

Gonadotropin-releasing hormone neurons are webbed intricately with the biological clock system and have been studied extensively for their participation in the circadian release of gonadotropins and sex steroids (23–25). There has been a growing interest in kisspeptin neurons, the upstream regulators of GnRH neurons, which are thought to possess similar circadian components (26, 27), achieving a synchronized operating mechanism in the HPG axis. Kisspeptin neurons are not inherently localized in high numbers in reproduction-related areas of the hypothalamus; they undergo a process of developmental maturation to attain their adult numbers and activity profile (28–30). Kisspeptin neuronal system is likely to “time” reproduction during development and aging by regulating circadian or estrus reproductive cycle as well as pulsatile GnRH/LH secretion.

## Neurochemical Properties of Kisspeptin

Kisspeptin belongs to the RF-amide peptide family. The propeptide, consisting of 145 amino acids is encoded by the *Kiss1* gene (31), which upon cleavage by the convertase enzyme, furin, generates the active form of kisspeptin, Kp54 (32). Shorter peptides such as Kp10, Kp13, and Kp14 are found in circulating levels in the placenta and result from the fragmentation of the unstable Kp54 (33, 34). Kisspeptin neurons adopt different functional roles in the AVPV and ARC as depicted by their opposing estrogenic response to ensure the inclusion of both a positive and negative feedback loop within the HPG axis, respectively (7, 35) (Figure 1).

Kisspeptin neurons in the AVPV are estrogen-sensitive, co-expressing high percentage of ERα type receptors (approx. 99% in rodents) and lower percentage of ERβ type receptors (approx. 31%) (7). Steroid-dependent activation of kisspeptin *via* ERα are imperative for the positive feedback response as depicted by the increased number of cells expressing *Kiss1* mRNA following estradiol treatment in intact female mice, and the lack of shift in firing pattern by estradiol in ERα knockout mice (36–38) (Figure 1). Peptides such as arginine vasopressin (AVP) (39) and gonadotropin-inhibitory hormone (GnIH) (40) and neurotransmitters including glutamate and gamma-aminobutyric acid (GABA) (41, 42) also regulate AVPV kisspeptin neurons (Figure 1). It was also reported that AVPV kisspeptin neurons co-express galanin (43–45) and dopamine (46).

As opposed to the AVPV, kisspeptin neurons in the ARC are sexually differentiated during the prepubertal stages and achieve stability both in terms of *Kiss1* expression and cell number during adulthood; latter attributed to the prevailing steroidal environment (47, 48). Furthermore, they co-express ERα, ERβ, and androgen receptors (7, 8), such that castrated/ovariectomized rodents exhibit increased *Kiss1* expression levels, which are suppressed upon exposure to testosterone and estrogen (7, 8) (Figure 1). Kisspeptin neurons also respond to GnIH (49) *via* GPR147 receptors (50, 51) (Figure 1).

### The Role of Kisspeptin in Reproduction

Parhar et al. were the first to elucidate the association between kisspeptin and GnRH, and the role of kisspeptin within the reproductive systems of non-mammalian vertebrates (52). Subsequently, similar findings were reported in mammalian species, including mice (53) and rats (6). Distribution studies demonstrated the presence of *Kiss1-R* in hypothalamic regions engaged in reproduction, such as the POA, ARC, and dorsomedial hypothalamus (DMH) (54, 55). The concentration of *Kiss1-R* in the POA was found to colocalize with GnRH neurons, with kisspeptin fiber projections evident in 40 and 10% of GnRH neurons in the POA of adult female and male mice, respectively (28, 52). Furthermore, the *in vivo* administration of kisspeptin triggers an acute release of GnRH, an effect that is abrogated in *Kiss1-R* knockout mice (56).

Loss of function of kisspeptin is detrimental to the HPG axis. *Kiss1*/*Kiss1-R* gene mutations are characterized by general infertility and cause abnormal gonadal development, delayed sexual maturation, and aberrant hormone secretion (57–59). Similar sexual irregularities and decreased serum gonadotropin levels are evident in GnRH-specific *Kiss1-R* knockout mice (60), endorsing kisspeptin as the prime effector of GnRH.

### Developmental Timing of Kisspeptin – A Checkpoint for Reproductive Functionality

The kisspeptinergic system develops dynamically throughout the mammalian lifespan until the start of the aging process. The maturation of the kisspeptin system begins with an increase in kisspeptin neurons from peripubertal stages in C57BL/6J mice (28) (Table 1). Kisspeptin cells are absent on postnatal day 10 (P10) and rapidly increase in number from P25 (28, 29). This increase in the neuronal population during development occurs concomitantly with both the formation of kisspeptin fiber appositions to GnRH neurons and *Kiss1-R* mRNA expression in GnRH neurons (28, 61) (Table 1). Connectivity between kisspeptin and GnRH neurons is barely noticeable before PN25 and slowly increases until puberty (28). The small number of kisspeptin cells evident during the prepubertal stage suggests that the kisspeptinergic system is “not yet-functional” and immature, an idea supported by the fact that during the same period GnRH neuronal activity is regulated by glutamatergic and GABAergic inputs (62).

**Table 1:** **Developmental changes in kisspeptin–GnRH system in female mice**.

	Postnatal (P0-30)	Puberty (P30-35)	Adult (8 weeks–1 year)	Aging (1 year)
Kisspeptin cell number in AVPV	No cells at P10 Cell number increases from P25	Adult level	–	Increase in morphologically irregular cells
GnRH neurons with kisspeptin fibers	Close apposition between kisspeptin fibers and GnRH cell bodies become apparent on P25	Rapid increase to reach adult level	–	?
GnRH neurons with Gpr54	40% of GnRH neurons express Gpr54 at P0, approximately 70% from P20	Adult level	–	?

*Adapted from Clarkson and Herbison (28), Herbison et al. (61), Clarkson et al. (30), and Zhang et al. (63)*.

Puberty is a fundamental stage, during which the kisspeptin system achieves its full functionality. Kisspeptin expression increases exponentially from the time of puberty until adulthood (30). A sevenfold increase in the number of *Kiss1* mRNA-expressing cells in AVPV of male mice is evident during the transition from puberty to adulthood, a pattern consistent with a fivefold increase in the number of kisspeptin expressing cells from PN25 to adulthood (64). However, these changes are exclusive to AVPV: the number of *Kiss1* mRNA-expressing cells remains unchanged in ARC (64). Evidence also exists to support the increased responsiveness of GnRH neurons to kisspeptin: 44% GnRH neuronal activity upon exposure to kisspeptin is evident during puberty, whereas 90% is evident during adulthood in male mice (28, 64). Interestingly, the pulsatile secretion of GnRH changes from one pulse every 90 min to one pulse every 30 min from the early postnatal to pubertal stages in male rats (65). The stability in the frequency of GnRH release marks the maturation of the HPG axis and suggests the onset of an active kisspeptin–GnRH stimulatory mechanism, which is required for a preovulatory GnRH/LH surge in females (66, 67).

Aging is a process that entails numerous degenerative processes and affects reproductive traits. In female mice, although the number of kisspeptin neurons remains unaffected during the aging process, specific cellular changes occur, including:

- decreased numbers of ERα-positive kisspeptin neurons,
- decreased kisspeptin neuronal activity at the time of LH surge, and
- morphological cellular changes in kisspeptin neurons featuring irregular shapes and atypical nucleus/cytoplasm (63) (Table 1).

A study by Ishii et al. showed that *Kiss1* mRNA and peptide expression in AVPV remains unchanged in middle-aged rats compared with young rats (68). The findings suggest that age-related reproductive neuroendocrine deficiencies originate from a loss of response of kisspeptin neurons in AVPV to estrogenic signals, therefore altering its secretory pattern and disrupting stimulation of the HPG axis (69). Aged rats (18–21 months old) exhibit reduced LH and FSH secretion, which coincides with decreased *GnRH* mRNA expression in the POA and reduced GnRH fiber projections to the median eminence (ME) (70–72).

## Integration of Rhythmicity within the Hypothalamic–Pituitary–Gonadal Axis – “Cycling” Reproduction

Several brain circuits converge to maintain the timely activation of the HPG axis: this maintenance is achieved by rhythmic cues originating mainly from the central biological clock located in the suprachiasmatic nucleus (SCN) (73, 74). The SCN is anatomically structured into a core and a shell, termed the ventrolateral (*vl*) and dorsomedial (*dm*) SCN, demarcated by neuropeptide composition (75). Vasoactive intestinal peptide (VIP) is the main neuropeptide released by most neurons of the *vl*SCN, which account for 10% of the total SCN, whereas neurons synthesizing AVP are localized in the *dm*SCN (76, 77). The *vl*SCN acts as the conductor of rhythmicity and transmits synchronizing cues to the *dm*SCN, which in turn amplifies the signal and conveys it to slave oscillators present in other brain regions and cellular entities (78) (Figure 1).

Among different hypothalamic nuclei of the HPG axis, the POA is densely innervated by VIP-ir fibers, with a subpopulation of GnRH neurons (±40%) expressing the cognate receptors for VIP, VPAC2/VIP2 (79) (Figure 1). This connectivity is further supported by the decrease in VIP-ergic innervations to the POA following lesions of VIP-positive neurons in the SCN (80). Importantly, the connectivity between the SCN to GnRH neurons is strengthened with an increase in VIP contacts to GnRH cells from prepubertal stages to adulthood, suggesting a circadian clock-aided maturation of the reproductive axis with the generation of estrous cyclicity and hormonal rhythms (81). Signals from the SCN are relayed to the AVPV through AVP fiber projections, as substantiated by anterograde tracing from the central clock (82). Studies have demonstrated expression of AVP-specific receptor, V1a, in kisspeptin neurons, and manipulation of AVP content in the brain elicits a time-dependent response of kisspeptin neurons (39) (Figure 1).

The DMH is also an important brain region for reproductive functionality, given its high content of GnIH neurons and co-expression of GPR147 receptors, specific to the GnIH peptide, in both kisspeptin and GnRH neurons (40, 50, 83–85) (Figure 1). The DMH receives extensive fiber projections from both regions of the SCN, although the majority of the fibers originate from the *vl*SCN (86). Exogenous administration of VIP triggers a decrease in GnIH cellular activity that is confined to the evening, mediating its time-specific modulation (87). Nonetheless, GnIH neurons do not co-express VIP receptors (87), suggesting that its neuronal activity is regulated by the circadian clock *via* alternate pathways, such as interneurons (Figure 1). On the other hand, it was recently demonstrated that GnIH inhibits VIP signaling in GnRH neuronal cell line, GT1-7, and inhibits VIP induced GnRH release from hypothalamic culture of female mice (88). Accordingly, GnIH neurons may modulate the activity of GnRH neurons in parallel with VIP, possibly to translate endogenous hormonal signals [estradiol: (89, 90) (Figure 1); melatonin: (84); glucocorticoid: (91, 92)]. Based on the fiber networks between the SCN and reproductive nuclei such as the POA, AVPV, ARC, and DMH, it is suggested that interactions among the central biological clock and reproductive neurons ensure optimal reproductive functionality (Figure 1).

### Disruption of the Circadian System and Its Effects on Reproductive Functionality

It is generally accepted that ablation of the SCN leads to reproductive incompetency by affecting subparts of the HPG axis such as interfering with diurnal variation of reproductive hormones (93). Females are more prone to this ablation as reported by a loss of ovulation, disruption of estrous cyclicity with desynchronized LH surge, and vaginal cornification induced by acyclic prolactin levels (94–96).

Circadian rhythmicity of SCN neurons is maintained by the transcriptional auto-regulatory loop between the clock genes and their products. Clock genes, such as *Period 1* (*Per1*) and *Period 2* (*Per2*), are transcriptionally activated by photic signals (97–99) and regulate the expression of *Brain and muscle ARNT-like 1* (*Bmal1*), which dimerizes with *Circadian locomotor output cycle kaput* (*Clock*) to enhance circadian transcriptional activity (100, 101). Additionally, *Per* and cryptochrome genes (*Cry*) are involved in the negative limb of the clock system by repressing CLOCK:BMAL1-induced transcriptional activity (102–104). Repression of the circadian machinery is also undertaken by genes such as *Rev-Erb*α (also known as *Nuclear receptor subfamily 1, group D, member 1*) (105, 106) and *Glycogen synthase kinase 3*β (107).

Global mutations, as listed in Table 2, generate stronger reproductive deficiencies as opposed to site- or neuron-specific gene alterations. *Clock* mutant female mice do not exhibit LH surges and normal estrous cyclicity (96). *Bmal1* knockout male mice exhibit lowered testosterone levels, accompanied by high serum LH concentrations (108). These results also indicate that the effects of clock-gene mutations on the HPG axis differ between the sexes, as suggested by gonadal and sex chromosome-dependent differences in the circadian system (109).

**Table 2:** **Reproductive deficiencies observed in clock genes mutants mice**.

Clock gene	Gender	Mutation	Reproductive deficiencies
Clock	Female	Clock^Δ19^	Irregular and lengthened estrous cycles, ↑ fetal reabsorption and term-pregnancy failures, interferes with coordinated release of GnRH, abnormal LH secretion patterns, affects maternal behavior, growth, litter size, and survival of pups (96, 110–112)
	Male	Clock^Δ19^	No significant difference in male fecundity (110, 112)
Bmal1	Female	Bmal1^−/−^	Infertile following sub-developed reproductive organs, abnormal estrous cycles, ↓ progesterone synthesis (108, 113–115)
	Male	Bmal1^−/−^	↑ LH levels, ↓ testosterone levels, impaired steroidogenesis, and accelerated reproductive aging (108, 115)
Per1/Per2	Female	Per1^−/−^ and Per2^−/−^	No signs of reproductive instability in young adult stages; mid-aged mutants have prolonged and acyclic estrous cycles (116)
	Male	–	No available literature on male rodents

*Bmal1, Brain and muscle ARNT-like 1; Clock, Circadian locomotor output cycle kaput; FSH, follicle-stimulating hormone; GnRH, gonadotropin-releasing hormone; LH, luteinizing hormone; and Per, period*.

### Rhythmicity of GnRH Neuronal Activity

Clock genes, such as *Bmal1, Per1* and *Per2*, exhibit rhythmic mRNA expression synchronous with oscillations in *GnRH* levels in GT1-7 cell line (117, 118). The intrinsic circadian molecular machinery of GnRH neurons is responsible for the mode of GnRH secretion, because a mutation in the Clock gene results in significant decrease of GnRH pulse frequency. On the other hand, overexpression of *Cry* gene increases GnRH pulse amplitude without changing pulse frequency (117). An *in vivo* study showed the sub-fertile attributes of GnRH-specific *Bmal1* knockout mice, characterized by irregular LH secretion while retaining normal reproductive processes, including estrous cycle (119). These results suggest that although GnRH neurons have intrinsic molecular timing machinery, GnRH neurons have to be properly regulated by other neurons to achieve regular GnRH/LH secretion.

The circadian regulation of GnRH neurons is two-tiered, because they also receive VIP-ergic afferents from the *vl*SCN and possess VPAC2 receptor (79). GnRH neuronal firing is activated in OVX + E mice by VIP during surge onset, but not in OVX mice. Administration of VIP receptor antagonist during surge peak decreases GnRH neuronal activity (120). An *in vitro* study using GT1-7 cells showed that GnRH neurons exhibit daily changes in GnRH levels and secretory patterns following stimulatory cues from VIP and kisspeptin (121). These results suggest that VIP can directly regulate circadian rhythm of GnRH neurons (Figure 1).

Gonadotropin-releasing hormone/luteinizing hormone release is also regulated by AVP. The circadian GnRH release coincided with circadian AVP release in coculture experiment of female rat POA and SCN (122). Administration of AVP into the POA of SCN lesioned female rat induced surge-like LH release (123). Interestingly, the effect of AVP administration to the POA in SCN-intact female rat was time-dependent. When AVP was administered during the second half of the light period, LH surge was induced in 30% of the animals; however, AVP had no effect when it was administered during the first half of the light period, indicating that AVP is part of the circadian regulatory machinery of LH surge (124). Intracerebroventricular (icv) injection of AVP on the afternoon of proestrus also induced LH surge in *Clock* mutant mice (125). Because AVP has no direct projection on GnRH neurons, these effect of AVP on GnRH/LH release may be mediated by AVPV kisspeptin neurons because SCN sends AVP fiber projections to AVPV and AVPV kisspeptin neurons express AVP-specific receptor, V1a (39, 82) (Figure 1). Effect of AVP on GnRH or LH release shown in *in vitro* (122) and *in vivo* (123) may also have been mediated by AVPV kisspeptin neurons because AVPV and POA regions are located closely in the brain.

### Rhythmicity of AVPV Kisspeptin Neuronal Activity

Kisspeptin mRNA in the AVPV peaks during the evening of proestrus in female rats, whereas kisspeptin mRNA in the ARC does not (126). AVPV kisspeptin neurons display rhythmic characteristics in ovariectomized female mice administered with constant estradiol, with peak expression occurring during late subjective day coincident with LH release. *Kiss1* rhythmicity only occurs in the presence of steroidal milieu: gonadectomised females are devoid of *Kiss1* rhythms, paralleled by a lack of LH rhythmicity (26). The circadian increase in *Kiss1* expression in the AVPV and the activation of GnRH cells were further shown to be dependent on ipsilateral neural input from the SCN (127).

A recent study showed the presence of circadian expression of *Per1* in AVPV kisspeptin neurons. Interestingly, *Per1* rhythm in the AVPV was estradiol-dependent (27). Noradrenaline (NA) is one of the modulators of GnRH release, and NA fiber terminals exist in close apposition to AVPV kisspeptin neurons. The use of Prazosin, an α1-adrenergic blocker, altered *Kiss1* mRNA expression and *Kiss1* contents, associated with the disruption in *Clock* and *Bmal1* expression in the POA (128), providing further evidence to the circadian regulation of kisspeptin signaling by clock genes.

Another trait endorsing rhythmicity of the kisspeptinergic system is the cyclic expression of *Kiss1-R* by GnRH neurons, which is dependent on an elevated steroidal environment, explaining its oscillating levels prior to the LH surge (129). This receptor expression pattern occupies a prime role in regulating the sensitivity of GnRH neurons to kisspeptin (39, 129), contributing to its cyclic secretion profile.

### Rhythmicity of ARC Kisspeptin Neuronal Activity

Ovariectomized rats with subcutaneous estradiol capsules were administered with kisspeptin or kisspeptin antagonist *via* bilateral intra-ARC or intra-POA cannulae or icv cannulae, and blood samples were collected for LH measurement *via* intravenous catheters. Administration of kisspeptin resulted in a dose-dependent increase in LH release. Although icv and intra-ARC administration of kisspeptin antagonist profoundly attenuated LH pulse frequency, intra-POA administration of kisspeptin antagonist did not affect pulsatile LH secretion (130). Dense kisspeptin fibers from ARC terminate at GnRH axons in the ME (44, 131, 132) (Figure 1). Accordingly, ARC kisspeptin may stimulate the frequency of pulsatile release of GnRH in the ME.

Kisspeptin neurons of ARC are referred to as KNDy neurons, because of their unique co-expression with neurokinin B (NKB) and dynorphin (Dyn) (133). The cellular activity of KNDy neurons is induced and repressed by NKB and Dyn, respectively, and ARC also possesses a subpopulation of NKB neurons that are not kisspeptin related and mediate direct actions on GnRH secretion (134). Central administration of Dyn inhibited multiple-unit activity (MUA) in the medial basal hypothalamus and pulsatile LH secretion, whereas NKB induced MUA and pulsatile LH secretion (135). These results suggest that ARC kisspeptin neurons regulate pulsatile GnRH/LH secretion acting with NKB and Dyn in the ARC.

## Concluding Remarks

It is thought that the generation of oscillations within cellular entities requires coordinated inputs from the SCN as well as an intrinsic circadian machinery (136, 137). As reviewed above, the cyclic reproductive functions also rely on regulatory cues originating from the SCN. Kisspeptin neurons exhibit their “timed” actions from their maturation to regulate GnRH/LH pulse and surge. These characteristic features of kisspeptin neuronal activity are in line with the influence of the central biological clock in imparting rhythmic cues to slave oscillators present in individual cells, and its coordinated entrainment of other elements of the HPG axis to ensure normal reproductive functions. It was shown that disruption of clock genes in GnRH neurons modifies the frequency and amplitude of GnRH pulse. Given the dearth of information on the role of circadian genes in the regulation of kisspeptin neurons, site-directed clock gene mutation study is imperative to understand the role of its intrinsic oscillatory mechanism in reproduction. It is also important to study how estradiol drives the oscillatory mechanism of kisspeptin neurons in females.

## Author Contributions

All authors listed, have made substantial, direct and intellectual contribution to the work, and approved it for publication.

## Conflict of Interest Statement

The authors declare that the research was conducted in the absence of any commercial or financial relationships that could be construed as a potential conflict of interest.
